# Arbuscular mycorrhizal strategy for zinc mycoremediation and diminished translocation to shoots and grains in wheat

**DOI:** 10.1371/journal.pone.0188220

**Published:** 2017-11-16

**Authors:** Abdelghafar M. Abu-Elsaoud, Nivien A. Nafady, Ahmed M. Abdel-Azeem

**Affiliations:** 1 Department of Botany, Faculty of Science, Suez Canal University, Ismailia, Egypt; 2 Department of Botany and Microbiology, Faculty of Science, Assiut University, Assiut, Egypt; Estacion Experimental del Zaidin, SPAIN

## Abstract

Mycoremediation is an on-site remediation strategy, which employs fungi to degrade or sequester contaminants from the environment. The present work focused on the bioremediation of soils contaminated with zinc by the use of a native mycorrhizal fungi (AM) called *Funneliformis geosporum* (Nicol. & Gerd.) Walker & Schüßler. Experiments were performed using *Triticum aestivum* L. cv. Gemmeza-10 at different concentrations of Zn (50, 100, 200 mg kg^-1^) and inoculated with or without *F*. *geosporum*. The results showed that the dry weight of mycorrhizal wheat increased at Zn stressed plants as compared to the non-Zn-stressed control plants. The concentrations of Zn also had an inhibitory effect on the yield of dry root and shoot of non-mycorrhizal wheat. The photosynthetic pigment fractions were significantly affected by Zn treatments and mycorrhizal inoculation, where in all treatments, the content of the photosynthetic pigment fractions decreased as the Zn concentration increased in the soil. However, the level of minerals of shoots, roots, and grains was greatly influenced by Zn-treatment and by inoculation with *F*. *geosporum*. Treatment with Zn in the soil increased Cu and Zn concentrations in the root, shoot and grains, however, other minerals (P, S, K, Ca and Fe) concentration was decreased. Inoculation of wheat with AM fungi significantly reduced the accumulation of Zn and depressed its translocation in shoots and grains of wheat. In conclusion, inoculation with a native *F*. *geosporum-*improves yields of wheat under higher levels of Zn and is possible to be applied for the improvement of zinc contaminated soil.

## Introduction

Heavy metals (HMs) naturally occurring in the environment are not the main source of risk; rather the industrial processing of these elements and their deposition into the environment due to this process [1]. HMs are mainly phytotoxic, either at all levels (e.g. Cd, Pb and As) or above certain threshold concentrations (e.g. Na, K, Cu, Zn, Co, Ca, Mg, Mn and Fe) and cause harmful effects to the environment, by affecting biomass and crop yields, soil fertility and, ultimately, human health [2–4]. Increase human health by minimizing the environmental risks posed by the accumulation of several toxic HMs in the soil (e.g., Cu, Cd, Pb, and Zn), accompanied by the high cost of cleaning soil contaminated by HMs [5]. Efforts have been promoted to develop new approaches for soil removal and cleaning of heavy metals by microfungi as plant symbionts with enhanced cleaning efficiency [5]. Zinc is an essential plant nutrient and is one of the most ubiquitous trace metals present in the soil. When present at excessive concentrations it is considered as a phytotoxic to plants [6]. Pollution of different ecological habitats by metals is already widespread in Egypt and around the world lead to increase toxicity of plants [7–9]. The transfer of heavy metals to plants through contaminated soil and/or irrigation water is the main route for animals and the human population with dangerous effects [10]. The metals and elements are nondegradable and subsequently accumulate in soils and plants [2, 11]. The exposure of humans to metals came through different pathways, like from soils to plants through the food chain [7]. The prediction of the transfer of metals from the soils of the shoot system through the radical tissues is important to be used in simulation models [12]. The root system is the main input step among plant tissues and its content is directly proportional to the bioavailability of HM levels in soils. The bioavailability of HMs fractions in the soil is largely conditioned by soil pH and determined by many factors like the magnitude of HMs, exposure time, presence or absence of nutrients and other chemical species [13, 14].

In 1996, WHO reported that human zinc poisoning included nausea, vomiting, diarrhea, fever and lethargy, and have typically been observed following the ingestion of 4–8 g of zinc. Kloke [15] recorded normal Zn content intervals in soils 3–50 (mg kg^-1^), while Nriagu [16] recorded the maximum permissible limits for Zn as 300 mg kg^-1^. Excess of 500 mg kg^-1^ in the soil interferes with the ability of plants to absorb other essential metals, such as iron and manganese [17]. The content of total Zn (mg kg^-1^) of Egyptian soil samples varied in their characteristics carried out by several researchers ranging from 18.3 to 176 mg kg^-1^ [18–20].

Mycoremediation (fungal remediation) is an evolutionary approach that can provide environmental benefits in addition to a cost-efficient alternative to other remediation methods [21]. Mycoremediation encompasses the use of fungi in the substantial or partial remediation of certain heavy metal contaminants in contaminated surface and groundwater, industrial wastewater, soil, sediment and sludge [22–24]. The Mycoremediation method is comparatively economical and requires expertise in the design of field projects with technical strategies and to develop a successful strategy of mycoremediation, the correct fungal species should be chosen to target a specific contaminant [25].

In order to bioremediate zinc, researchers have attempted to look for efficient plant species with the ability to accumulate zinc from soils [26, 27]. Macrophytes have been used during the last two decades for the removal of metals [3, 27–30]. Several species of plants manage to coexist and survive in such contaminated soils is a key issue in ecology [31, 32]. Glomeromycota is a group of soil microfungi that form a symbiotic relationship with the root system of terrestrial plants [33]. More than eighty percent of terrestrial plants are in a symbiotic relationship with arbuscular mycorrhizal (AM) fungi [34, 35]. As a consequence of AM colonization, fungi help nourish plants with water and nutrients, promote host resistance to biotic and abiotic stress, and consequently improve overall plant productivity and health [36–40]. The mycorrhizal colonization technique has been carefully introduced in agriculture to provide benefits to cultivated plants, which involves improving plant metabolism, producing secondary metabolites and helping to improve and maintain an optimal soil texture [41]. Inoculation of the Indian wheat genotype to arbuscular mycorrhizal fungi in sandy loam soil indicated better phosphorus absorption, growth and yield consequences in mycorrhizal plants and efficiently saved fertilizer inputs and phosphorus utilization [42]. A meta-analysis on wheat inoculation by arbuscular mycorrhizal fungi (AMF), comprising 38 published field trials with 333 observations, revealed that AM fungi can benefit wheat crop growth and yield through increased biomass, grain N content, Zn uptake and grain yield by 20% [43].

Mycorrhizal fungi have also been supplemented to fertilizers in order to increase growth and productivity of crops [44]. Several investigations on plants inoculated with mycorrhizae under the impact of bulk metals have demonstrated that host plants were protected against HM stress and toxicity [45, 46]. Mycorrhizal colonization facilitates phytoremediation by promoting the growth of hyperaccumulating plants [40, 47, 48]. However, because of the distinctive physico-chemical characteristics of metal or metal oxide nanoparticles, the impact of these materials could have an inhibitory effect on plant colonization by AM fungi [49].

This study aims to examine the physiological role of a native *Funneliformis geosporum* on the uptake, translocation of Zn and other nutrients in the survival of *Triticum aestivum* as one of the most economical plants in the world.

## Materials and methods

### Sampling, soil analysis and isolation of AMF

Five rhizospheric soil samples were collected randomly form different crops namely: *Hibiscus sabdariffa* L., *Zea mays* L., *Lycopersicum esculentus* L., *Cicer arietinum* L. and *Triticum aestivum* L. from Assiut Governorate, Upper Egypt (26.8333 to 27.6166 N and longitude of 30.6500 to 31.5833 E) for the isolation of AM fungi. Samples were kept in a clean sterile polyethylene bags, labeled and sent to the laboratory within one hour for analysis. The particle size distribution was carried out using the screening method [50]. The content of organic matter was estimated by the method of Walkely and Black [51]. Total calcium carbonates were determined volumetrically by Collin's Calcimeter [52] and chemical characteristics [Cations (Na^+^, K^+^, Mg^2+^, Ca^2+^), anions (Cl^-^, HCO_3_^-^, PO_4_^3-^, SO_4_^2-^)], EC and pH were determined in 1:2 soil-water extract according to Jackson [51] and Page [53]. The concentrations of heavy metals (Zn and Fe) were estimated using the total adsorptive metals technique based on USEPA [54] using atomic spectrophotometer (Model PYE-UNICAM SP9, England).

For isolation and examination of AMF from rhizospheric soils, wet sieving and decantation technique was used [55]. AMF taxa were identified according to the relevant keys [56–59] using morphological characteristics of hyphae, bound hyphae, chlamydospores, azygospores and sporocarp. For root cleaning and staining of mycorrhizae, Philips and Hayman technique was applied [60, 61].

### Plant material

Grains of *Triticum aestivum* L. cv. Gemmeza-10 was sterilized for 1 minute in 75% ethanol and then immersed for 3 minutes in sterile distilled water. The seeds were allowed to dry overnight and then grown in plastic pots (35 cm high x 26 cm wide).

### Experimental design

The experiment was carried out in a greenhouse at Department of Botany and Microbiology, University of Assiut, during the wheat growing season from December to February under ambient light (89.5–85.8 hours of sunshine), temperature (17–26°C) and humidity (43–62%). Soils for mycorrhizal production and artificially polluted experiment were collected from the Botanical garden of Department of Botany and Microbiology, University of Assiut. Soil of loam and sand mixture (2: 1, w/w) was steamy sterilized (121°C for 1 h, for 3 alternate days) packed in 5 Kg soil pot^-1^ and set in a completely random design with three replicates. For artificial pollution, three levels of Zn (50, 100 and 200 mg kg^-1^) were added in the form of ZnCl_2_. Mycorrhizal (M) and non-mycorrhizal (NM) pots were irrigated with tap water when required.

### Mycorrhizal inoculum

*Funneliformis geosporum* (Nicol. & Gerd.) Walker & Schüßler, as a dominant taxon in wheat rhizospheric soils under study,was selected. The selected isolate (*F*. *geosporum*) was maintained on the onion roots (*Allium cepa* L.) as host plants [62] cultured for three months before the start of the experiment. The inoculum suspension was prepared by wet sieving technique including: colonized root fragment, hyphae and 1000 spores pot^-1^. The freshly prepared suspension was inoculated 3 cm below the wheat grains, while non-mycorrhizal (NM) control group received a similar volume of autoclaved suspensions.

### Growth parameters and grain yield

Three plants were harvested in the late vegetative stage (45 days after sowing) of each pot with a total number of 9 plants. The plants were separated into shoots and roots and a part of the roots and fresh shoots were immediately frozen in liquid nitrogen for analysis. The dry weight of the plant was estimated by placing the samples in an oven at 60°C until obtaining a constant dry weight [63]. In addition, some of wheat were harvested at the ripening stage to record the yield component data, which included: plant height (cm), days to flowering, days to maturity, spike length (cm), grain yield (g plant^-1^), biological yield (g plant^-1^), weight of 100 grams (g), number of grains / spike [64].

### Estimation of photosynthetic pigments

Pigment fractions (chlorophyll-a, chlorophyll-b, and carotenoids) were estimated using the spectrophotometric method as recommended by Lichtenthaler [65].

### Mineral analysis

Three individual randomly selected plants/replica were separated into shoots, roots, and grains, and oven dried at 70°C for two days to determine the elemental concentrations (Zn, P, S, K, Ca, Fe, Cu) using JEOL JSM -5400 LV SEM supplied with a Tracor Northern 5200, energy dispersive X-ray (EDX) analysis system at Electronic Microscope Unit, University of Assiut. The sample powder was analyzed by using EDX unit attached to SEM expressed as an average of three points [66, 67].

The zinc efficiency was evaluated based on three different characteristics according to Yang et al. [68], the bioaccumulation factor (BCF) was calculated according to the following equation [68]:

$$BCF=\frac{C_{Zn-above\;ground}}{C_{Zn-Soil}}$$

In this expression, C_Zn_ -aboveground (mg kg^−1^) is the Zn concentration estimated in the aboveground parts of the plant including grains, while C_Zn_-soil (mg kg^−1^) is the Zn concentration in the soil.

Total Zn metal uptake in wheat was calculated as follows [68]:

$$Metal\;uptake\;(mg\;{pot}^{-2})=C_{metal\;concentration\;in\;plant\;tissue}\;(mg\;{Kg}^{-1})$$

$$\times W_{plant\;dry\;weight}\;(Kg\;{plant}^{-1})\;\times n_{number\;of\;plants}$$

The phytoextraction efficiency (%) of *T*. *aestivum* was calculated using the following equation:

$$Phytoextraction\;efficiency\;(\%)=\frac{C_{Zn\;in\;plant\;tissue}\;(mg\;{kg}^{-1})\times W_{plant\;dry\;weight}\;(kg\;{pot}^{-1})\times n_{number\;of\;plants}}{C_{Zn-soil}\times 5\;Kg\;{pot}^{-1}}$$

### Determination of mycorrhizal colonization rate

Root segments (2 cm) were stained with trypan blue (0.5% w / v) according to the Philips and Hayman method [60]. The intensity of root mycorrhizal colonization (M%), mycorrhizal frequency (F%) and root arbuscular frequency (A%) was calculated according to Trouvelot et al. [69] using the MYCOCALC program [70].

### Determination of lipid peroxidation

Lipid peroxidation was determined based on malondialdehyde (MDA) formation using thiobarbituric acid reaction modified after Rao and Sresty [71].

### Statistical analyses

Statistical analyses were performed using the IBM-SPSS statistical software for Mac OS version 23. One and two-way analysis of variance (ANOVA) were performed to assess the differences between the various treatment groups. Multiple Duncan’s multiple range comparisons were also performed. All experiments were performed in triplicates.

## Results

Various physicochemical characteristics of rhizospheric soil samples collected from open fields were analysed and presented in Table 1. The soil texture was clay with a pH ranges from 7.90 to 8.80. The mean electrical conductivity (EC) was 1.94±0.09 mmhos.cm^-1^ with a highest absolute value at site no. 5 soil with 2.04 mmhos.cm^-1^ and the lowest absolute value at site no. 4 soils was 1.82 mmhos.cm^-1^. The mean value of organic matter (OM) was 0.94% ±0.12. The mean concentration of available P in the soil samples was 13.44±2.56 mg kg^-1^, while and the mean concentration of Zn was 1.03±0.39 mg kg^-1^.

**Table 1:** pH values, electrical conductivity (EC dsm^-1^), cations and anions contents (mg/100 g), heavy metal contents (Fe and Zn mg kg^-1^), phosphorus contents (P; mg kg^-1^), the percentage of organic matter (OM%) and soils texture of 5 sites of cultivated soils.

Soil Parameter	Sampling sites						ANOVA	
	Site 1	Site 2	Site 3	Site 4	Site 5	Mean ± SD	F-ratio	*p*-value
**pH**	8.07	8.09	8.80	8.23	7.90	8.22 ± 0.35	2.0	0.177
**EC**	2.01	1.89	1.92	1.82	2.04	1.94 ± 0.09	13.5*	<0.001
**Ca**^**++**^	0.54	0.59	0.51	0.41	0.28	0.47 ± 0.12	0.3	0.878
**Mg**^**++**^	0.35	0.53	0.47	0.32	0.51	0.44 ± 0.10	0.1	0.965
**Na**^**+**^	3.29	3.49	2.98	3.19	2.79	3.15 ± 0.27	1.3	0.332
**K**^**+**^	0.11	0.08	0.09	0.07	0.09	0.09 ± 0.01	0.0	1.000
**Cl**^**-**^	0.59	0.42	0.87	1.17	1.32	0.87 ± 0.38	2.3	0.128
**HCO**_**3**_^**+**^	0.20	0.21	0.31	0.25	0.35	0.26 ± 0.06	0.1	0.994
**SO**_**4**_^**--**^	1.62	2.87	3.09	1.64	3.21	2.49 ± 0.79	10.3*	0.001
**Fe**^**++**^	8.29	8.50	6.73	8.25	6.72	7.70 ± 0.89	13.5*	<0.001
**Zn**	0.59	1.34	1.27	1.31	0.62	1.03 ± 0.39	2.6	0.103
**P**	12.73	16.93	9.82	14.09	13.63	13.44 ± 2.56	109.7*	<0.001
**OM%**	0.95	1.08	0.85	1.03	0.79	0.94 ± 0.12	0.3	0.886
**Texture**	Clay	Clay	Clay	Clay	Clay		--	--

Differences between sites were assessed by one-way ANOVA were performed

* Significant for *p*<0.05

Analysis of physicochemical properties of loamy sand mixture used in greenhouse experiment showed that texture was limed, pH of 7.30±0.12, EC was 3.43±0.35 mmhos.cm^-1^, OM, total CaCO_3_ and total N were 14.8±0.62, 14.3±1.27 and 1.48±0.11 g kg^-1^ soil, respectively. The concentration of available P was 140±18.03 mg kg^-1^ soil and Zn was 1.68±0.21 mg kg^-1^.

Identified taxa of AM fungi, spore density and colonization rate were recorded and presented in Table 2. Totally, 14 AMF morphotypes were recovered from different plant species. Reported taxa were belonged to class Glomeromycetes belongs to, three orders (Glomerales, Diversisporales, and Archaeosporales). All the isolates were distrubited infive families (Glomeraceae, Gigasporaceae, Acaulosporaceae, Entrophosporaceae and Paraglomeraceae). A total of 425 spores and sporocarps were recovered from the 5 collected rhizospheric soil samples. The abundance of AM fungal colonization rate was widely varied from 62 to 90%, with the highest being recorded at site No. 3 cultivated with *Lycopersicum esculentus* L. and the maximum mean spore density also recorded at site No. 3 (142 spores/ 100 g of soil). The most common species was *F*. *mosseae* from which it was recorded at all sites, followed by *F*. *geosporum* (recorded at 4 sites out of five) (Table 2). *Funneliformis geosporum* (Nicol. & Gerd.) Walker & Schüßler, as a most abundant native taxon in wheat rhizospheric soil was selected for further investigation.

**Table 2:** Distribution, root colonization (%) and spore density (The number of spores in 100 g soil) of AMF in 5 sites of cultivated soils.

Site no.	Plant species	Root Coloniza-tion (%)	AMF	Spore density (SD)
1	*Hibiscus sabdariffa* L.	64	*Funneliformis mosseae* (T.H. Nicolson & Gerd.) C. Walker & A. Schüßler	14
			*Rhizophagus antarcticus* (Cabello) C. Walker	6
			*Glomus caesaris* Sieverd. & Oehl	11
			*Paraglomus bolivianum* (Sieverd. & Oehl) Oehl & G.A. Silva	10
			*Rhizophagus clarus* (T.H. Nicolson & N.C. Schenck) C. Walker & A. Schüßler	17
2	*Zea mays* L.	*73*	*Acaulospora laevis* Gerd. & Trappe	17
			*Acaulospora rehmii* Sieverd. & S. Toro	3
			*Acaulospora thomii* Błaszk.	19
			*Acaulospora tuberculata* Janos & Trappe	7
			*Funneliformis geosporum* (T.H. Nicolson & Gerd.) C. Walker & A. Schüßler	6
			*Funneliformis mosseae* (T.H. Nicolson & Gerd.) C. Walker & A. Schüßler	21
			*Rhizophagus clarus* (T.H. Nicolson & N.C. Schenck) C. Walker & A. Schüßler	13
3	*Lycopersicum esculentus* L.	90	*Acaulospora laevis* Gerd. & Trappe	24
			*Acaulospora splendid* Sieverd., Chaverri & I. Rojas	11
			*Entrophospora infrequens* (I.R. Hall) R.N. Ames & R.W. Schneid.	14
			*Funneliformis geosporum* (T.H. Nicolson & Gerd.) C. Walker & A. Schüßler	19
			*Funneliformis mosseae* (T.H. Nicolson & Gerd.) C. Walker & A. Schüßler	28
			*Glomus caesaris* Sieverd. & Oehl	7
			*Gigaspora gigantea* (T.H. Nicolson & Gerd.) Gerd. &Trappe	11
			*Rhizophagus clarus* (T.H. Nicolson &N.C. Schenck) C. Walker & A. Schüßler	17
			*Scutellospora armeniaca* Błaszk.	11
4	*Cicer arietinum* L.	62	*Acaulospora tuberculata* Janos & Trappe	6
			*Entrophospora infrequens* (I.R. Hall) R.N. Ames & R.W. Schneid.	19
			*Funneliformis geosporum* (T.H. Nicolson & Gerd.) C. Walker & A. Schüßler	15
			*Funneliformis mosseae* (T.H. Nicolson & Gerd.) C. Walker & A. Schüßler	23
			*Rhizophagus clarus* (T.H. Nicolson & N.C. Schenck) C. Walker & A. Schüßler	8
			*Paraglomus bolivianum* (Sieverd. & Oehl) Oehl & G.A. Silva	9
5	*Triticum aestivum* L.	80	*Funneliformis geosporum* (T.H. Nicolson & Gerd.) C. Walker & A. Schüßler	18
			*Funneliformis mosseae* (T.H. Nicolson & Gerd.) C. Walker & A. Schüßler	13
			*Gigaspora gigantea* (T.H. Nicolson & Gerd.) Gerd. &Trappe	4
			*Rhizophagus clarus* (T.H. Nicolson & N.C. Schenck) C. Walker & A. Schüßler	15
			*Scutellospora armeniaca* Błaszk.	9
Total				425

**Fig 1:**
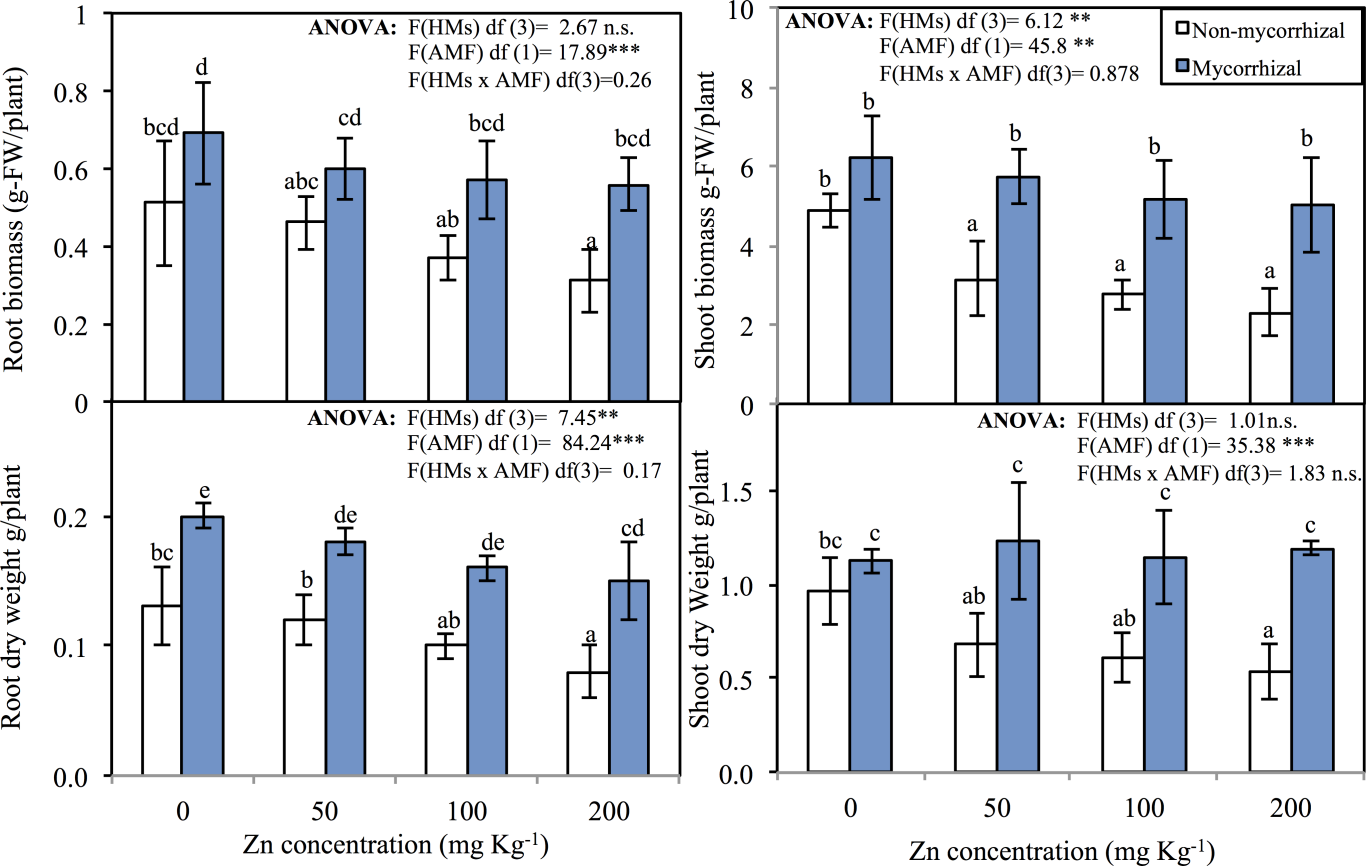
**Fresh and dry weights (g plant**^**-1**^**) of roots (A, B) and shoots (C, D) of mycorrhizal and non-mycorrhizal wheat affected by Zn treatments. The data represented are an average of five replicates; error bars represent the standard error for means.** ANOVA was carried out to evaluate differences between treatment groups, followed by multiple-rank comparisons of Duncan. Media with similar letters are not significantly different according to Duncan's multiple range comparisons.

The growth rate of *T*. *aestivum* plants expressed as g plant^-1^ of fresh or dry weight of roots or shoots (Fig 1A–1D). The data are shown in Fig 1 indicated that the treatments with Zn of 200 mg kg^-1^ significantly (*p*<0.05) reduced the fresh and dry weights of the roots as compared to the non-mycorrhizal (NM) and mycorrhizal (M) control (Fig 1A and 1B). In non-mycorrhizal plants, a significant reduction in root fresh weight was recorded at a higher Zn concentration, which decreased in the root from 0.51 (g plant^-1^) at the control level to 0.31 (g plant^-1^) at 200 mg kg^-1^ Zn and from 4.86 at the control level to 2.31 (g plant^-1^) at 200 mg kg^-1^ Zn (Fig 1C and 1D). Inoculation of wheat with AM fungus stimulated the shoot growth, however, this growth stimulation was not significant at compared with the control. The shoot growth at different zinc levels showed a significant effect (*p*<0.05) of AM fungus inoculation (Fig 1C and 1D). All concentrations of Zn exerted an inhibitory effect on the yield of roots and shoot dry weights of non-mycorrhizal plants; however, inoculation with AM fungi increased root dry weight at different zinc levels (Fig 1).

Wheat yield in response to mycorrhizal inoculation was monitored and presented in Table 3. Two yield parameters, days to maturity and flowering, of mycorrhizal wheat under Zn treatments were significantly lower than their corresponding non-mycorrhizal counterparts (Table 3). The mycorrhizal inoculated wheat untreated with Zn bloom after 62.78±0.7 days and maturity after 103.22±1.3 days, while, non-Zn treated plants without mycorrhization require 65.89±2.4 and 111.33±0.8 days for flowering and maturity, respectively. At the high level of Zn (200 mg kg^-1^), the non-mycorrhizal wheat required 70.56±2.6 and 119.33±3.0 days for flowering and maturity, respectively, whereas in mycorrhizal counterparts they required 64.33±0.8 and 109.56±1.1 days. In wheat treated with non-mycorrhizal Zn, plant height was significantly lower (*p*<0.05) than that of mycorrhizal plants treated with Zn. The plant height of wheat varied from 62.78 to 52.00 cm in NM wheat treated with 0 to 200 mg kg^-1^ Zn (Table 3).

**Table 3:** Yield parameters of wheat in response to mycorrhizal inoculation grown under different levels of Zn. Data were presented as the mean of three replicates followed by the standard deviation (mean ± SD).

Treatments		Days to flowering	Days to maturity	Plant height (cm)	Spike length (cm)	Grain yield (g plant^-1^)	Biological yield (g plant^-1^)	1000-grain weight (g)	Number of grains per spike
Zn (mg kg^-1^)	Inoc.								
**0**	**NM**	**65.9**±2.4^***bc***^	**111.3**±0.8^***abc***^	**62.8**±2.2^***cd***^	**8.9**±0.3^***bc***^	**10.2**±1.3^***bc***^	**17.6**±1.2^***ab***^	**26.7**±3.3^a^	**35.0**±2.3^***a***^
	**M**	**62.8**±0.7^***a***^	**103.2**±1.3^***ab***^	**68.6**±3.2^***e***^	**9.7**±0.5^***c***^	**16.2**±1.3^***e***^	**20.7**±1.1^***c***^	**36.2**±4.0^b^	**48.7**±1.7^***d***^
**50**	**NM**	**68.1**±0.7^***c***^	**115.2**±0.3^***d***^	**58.8**±3.8^***bc***^	**8.9**±0.5^***bc***^	**9.02**±1.7^***c***^	**15.7**±2.5^***bc***^	**28.7**±3.1^a^	**30.4**±2.8^***a***^
	**M**	**63.9**±0.8^***ab***^	**105.2**±2.5^***a***^	**71.6**±2.4^***e***^	**10.1**±0.1^***c***^	**15.5**±0.9^***e***^	**24.0**±0.3^***d***^	**34.1**±0.9^***b***^	**44.7**±5.0^***cd***^
**100**	**NM**	**69.7**±1.1^***c***^	**117.1**±2.3^***cd***^	**53.8**±1.9^***ab***^	**8.3**±1.4^***ab***^	**8.74**±0.6^***ab***^	**13.1**±1.2^***a***^	**26.8**±5.1^***a***^	**22.8**±0.9^***a***^
	**M**	**64.9**±0.8^***ab***^	**108.8**±1.1^***bc***^	**66.1**±2.3^***de***^	**10.0**±0.5^***c***^	**13.9**±1.1^***de***^	**23.3**±0.5^***d***^	**35.5**±2.3^***b***^	**42.6**±2.4 ^***cd***^
**200**	**NM**	**70.6**±2.6^***c***^	**119.3**±3.0^***d***^	**52.0**±0.8^***a***^	**7.3**±0.6^***a***^	**7.8**±1.0^***a***^	**11.7**±0.1^***a***^	**20.4**±0.8^***a***^	**16.0**±0.9^***bc***^
	**M**	**64.3**±0.8^***ab***^	**109.6**±1.1^***b***^	**68.9**±5.3^***e***^	**9.9**±0.4^***c***^	**12.1**±1.3^***d***^	**25.7**±1.9^***d***^	**33.9**±1.8^***b***^	**40.2**±1.4 ^***ab***^
**Analysis of variance (ANOVA)**									
**F**_**Zn**_		12.21*	22.89*	5.92**	1.87^NS^	3.83*	14.07*	23.59*	52.41**
**F**_**AMF**_		39.91**	37.34**	92.06**	32.02**	104.59**	111.74**	48.12**	121.35**
**F**_Zn_*_AMF_		20.53*	13.27*	3.41*	11.91*	6.86**	7.41**	32.34*	35.21**

One-way ANOVA was performed for each Zn concentration. Means with the same letter and are not significantly different according to Duncan multiple range comparisons. Two-way ANOVA was used to determine the influence of the concentration of AMF and Zn

NS: non—significant (*p*>0.05)

*—significant (*p*<0.05)

**—highly significant (*p*<0.01).

The length of the spikes, number and weight of the grains of wheat had increased by mycorrhizal association under different Zn-treatments. The effect of treatments with Zn on the number of grains/spike or their weights (weight of 1000 grains) was more pronounced at high Zn level (200 mg kg^-1^), where the number of grains spike^-1^ reduced to 16±0.9 grains and weight of 1000 grains reached 20.40±0.8 g. The results showed that inoculation of wheat with native AM taxon showed a positive effect on the number of grains/spikes, grain yield, and grain weight. The maximum number of grains/spike (48.78±1.7 grains) recorded at the mycorrhizal (M) control. Data on the 1000 grains weight and grain yield presented in Table 3 showed that the weight of 1000 grains decreased from 36.25±0.4 (at the M control) to 33.96±1.8 grains (at 200 mg kg^-1^ Zn) of mycorrhizal wheat. Similarly, the grain yield decreased from 16.17 in mycorrhizal (M) control plants to 12.09 g plant^-1^ at M wheat treated with 200 mg kg^-1^ Zn (Table 3). The biological yield (g plant^-1^) decreased in NM wheat at different Zn-treatments (0–200 mg Kg^-1^), however it was significantly (*p*<0.05) increased after inoculation with *F*. *geosporum* as shown in Table 3. The maximum biological yield (25.7±1.9 g plant^-1^) was reported in M wheat at 200 mg kg^-1^ Zn, while the minimum biological yield (11.7±0.1 g plant^-1^) was reported in NM wheat at 200 mg kg^-1^ Zn.

**Fig 2:**
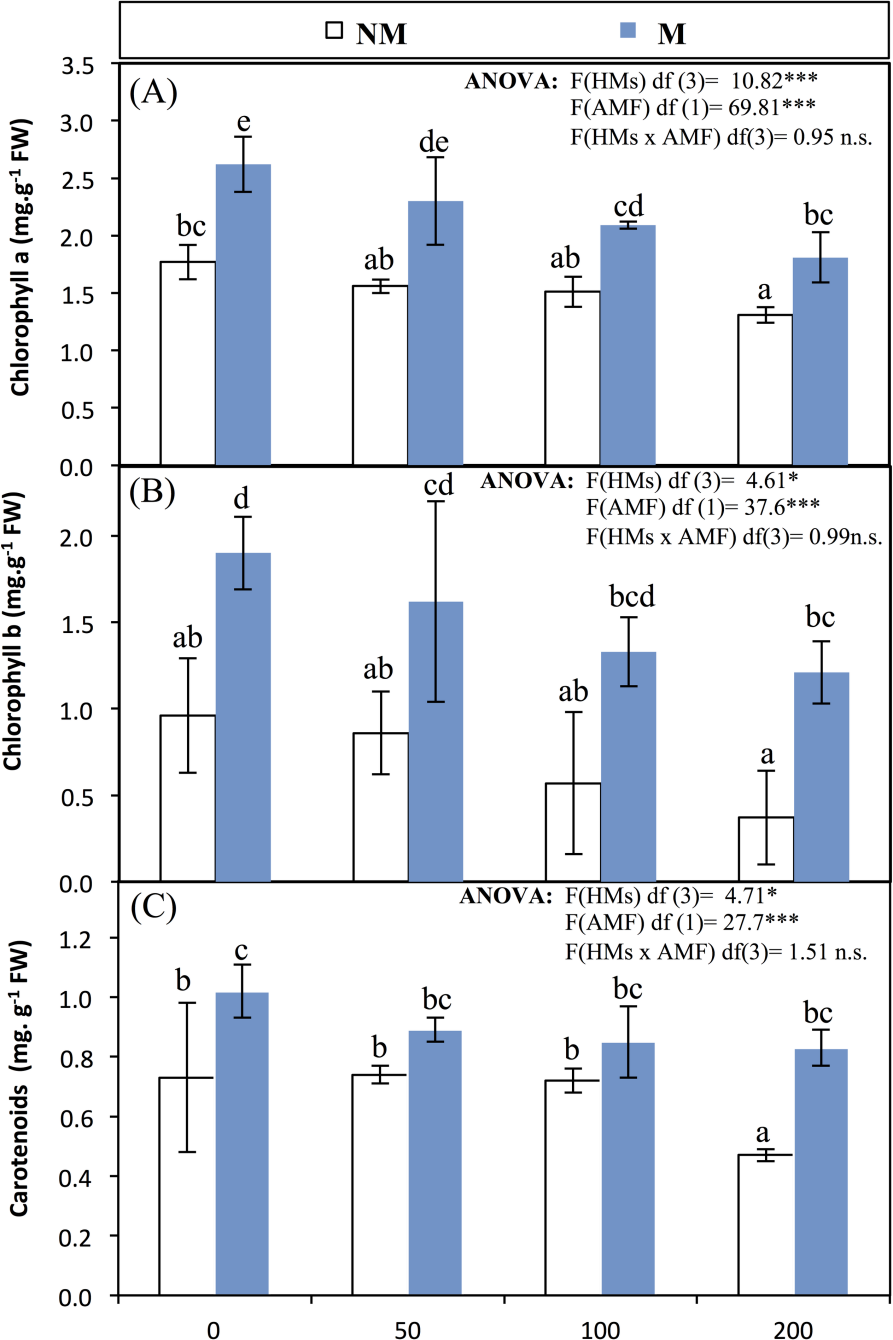
**(A) Chlorophyll-a, (B) Chlorophyll-b, (C) Carotenoid pigment content as mg g**^**-1**^ **FW of leaves of mycorrhized and non-mycorrhized wheat grown at different levels of treatments with zinc are an average of five replicates, the error bars represent the standard error for the means.** ANOVA was performed to assess differences between treatment groups, followed by Duncan's multiple-rank comparisons. Media with similar letters are not significantly different according to Duncan's multiple range comparisons.

The content of photosynthetic pigments (Chl-a, Chl-b, and carotenoids) of mycorrhizae and non-mycorrhizae in leaves of wheat are presented as mg g^-1^ of fresh leaf weight as represented in Fig 2A–2C. The present study shows that the fractions of photosynthetic pigments are significantly affected by the treatments with Zn and inoculation of mycorrhizae. In general, with all treatments, the content of the photosynthetic pigment fractions decreased as the Zn concentration increased in the soil. However, the content of photosynthetic pigments of leaves of M wheat was significantly higher (*p*<0.05) than that of NM ones, of which at control levels of 5.53±0.46 to 3.46±0.45 (mg g^-1^ FW) in M and NM leaves wheat, respectively. Carotenoid biosynthesis significantly (*p*<0.05) reduced by Zn-treatments in NM wheat, while non-significant increase was recorded in M wheat under different Zn levels (Fig 2C). The amount of chlorophyll-a, chlorophyll-b and carotenoids decreased under all Zn-treatments. This effect was more pronounced in the presence of 100 and 200 mg kg^-1^ Zn.

EDX analysis showed that the concentration of Zn in wheat’s root, shoots, and grains was linearly correlated with soil Zn concentration (Table 4). However, the rate of Zn accumulation decreased in mycorrhizal wheat (shoot, root, grains). The different plant organs exhibited a different pattern of accumulation, especially at higher levels of Zn. The highest concentration of metal was recorded in the shoots at 100 mg kg^-1^ Zn. Inoculation with AMF significantly reduced the accumulation of metals. Significant differences (*p*<0.05) were observed in the accumulation of zinc in NM and M wheat revealed by one-way ANOVA at each concentration of Zn in the soil. The concentration of zinc in roots increased from 6.42±0.67% in NM control to 47.91±5.96% in NM 200 mg kg^-1^ Zn, respectively. Statistical analysis showed that AM fungi significantly (*p*<0.05) induced a lower accumulation of Zn in wheat roots.

**Table 4:** Mineral content in shoots, roots, and grains of wheat grown under different levels of zinc. The data represented are an average of three replicates ± standard deviation.

Plant Tissue/ Heavy metals treatment (mg kg^-1^)		AM	Mineral concentration (%)						
			P	S	K	Ca	Fe	Cu	Zn
Root	0	NM	2.87±1.86 ab	3.14±0.28 c	11.13±0.17 cd	18.83±0.24 b	54.25±3.31 de	3.36±0.65 a	6.42±0.67 a
		M	5.57±1.54 c	2.88±0.56 c	12.72±3.03 d	22.97±9.26 b	42.56±6.65 c	7.47±6.10 ab	5.83±3.39 b
	50	NM	3.11±1.02 a	0.17±0.08 a	7.47±2.83 bc	1.83±0.15 a	20.92±0.65 b	35.55±2.26 d	31.33±2.04 d
		M	8.62±1.58 ab	1.37±0.21 b	10.05±3.16 cd	17.94±2.33 b	54.52±3.28 e	3.39±1.13 a	4.11±0.41 ab
	100	NM	2.22±2.62 ab	1.21±1.58 ab	4.87±1.76 ab	5.66±2.27 a	6.66±3.57 a	34.55±4.55 d	46.56±2.66 e
		M	7.09±1.82 bc	0.23±0.08 a	5.48±0.25 ab	6.77±0.45 a	48.59±5.56 d	15.59±2.70 c	16.24±2.39 c
	200	NM	1.72±0.57 a	0.17±0.06 a	1.89±0.09 a	8.35±2.24 a	2.62±0.20 a	37.00±3.62 e	47.91±5.96 e
		M	10.56±1.51 c	0.06±0.04 a	4.44±2.98 a	6.39±3.65 a	47.91±2.74 de	13.11±3.22 bc	17.74±1.40 c
	Two-Way ANOVA								
	All (df = 7)		15.34***	12.71***	9.64***	22.12***	66.99***	63.46***	66.42***
	HM (df = 3)		8.39***	26.31***	21.26***	38.40***	21.59***	50.48***	67.08***
	AM (df = 1)		65.40***	0.03 n.s.	2.00 n.s.	24.06***	194.63***	194.37***	157.64***
	HM*AM (df = 3)		5.60**	3.33*	0.57 n.s.	5.19*	65.37***	32.67***	35.35***
Shoot	0	NM	5.80±3.14 b	5.80±1.84d	28.13±3.76 c	20.88±3.72 b	24.78±6.88 a	3.03±0.86a	11.58±0.3 a
		M	6.41±1.43 c	6.12±0.69d	20.50±0.36 a	26.79±10.8 d	25.90±4.65 a	4.18±1.11a	10.09±5.6 ab
	50	NM	4.21±1.69 a	1.33±0.66a	8.02± 0.68 a	2.45±0.52 ab	10.67±1.01 a	36.65±5.58c	36.66±3.58 d
		M	16.96±2.09 c	5.85±1.30cd	17.63±6.16 a	15.13±2.52 d	20.75±0.14 a	9.21±3.95a	14.46±0.42 b
	100	NM	2.25±0.20 a	0.32±0.15a	4.42± 1.11 a	0.71±0.74 a	5.30±2.54 a	38.11±5.15d	48.88±5.24 e
		M	12.41±1.97 c	4.18±0.82b	21.81±3.57 b	4.54±2.41 ab	18.81±2.40 b	20.0 ±8.27b	18.91±6.86 c
	200	NM	2.13±1.92 a	1.01±0.39 a	6.98± 1.11 a	3.5±0.92 ab	7.38±2.74 a	33.02±2.87c	45.98±3.1 d
		M	12.47±4.06 b	4.55±1.07 b	16.10±1.39 b	11.23±6.3 c	15.59±7.12 ab	19.18±8.63b	20.89±5.7 c
	Two-Way ANOVA								
	All (df = 7)		21.75***	30.41***	37.60***	70.67***	4.02**	129.24***	405.82***
	HM (df = 3)		7.95**	35.35***	25.60***	54.21***	2.58n.s.	156.38***	733.29***
	AM (df = 1)		113.26***	61.78***	0.67n.s.	228.65***	4.23 n.s.	301.332***	1068.5***
	HM*AM (df = 3)		5.06*	15.02***	61.90***	34.48***	5.39*	44.47***	157.46***
Grain	0	NM	6.90±0.79abc	2.88±0.56 ab	12.72±3.03 a	32.9±19.9 bc	31.23±5.74 b	10.8±5.99 a	5.83±3.39 bc
		M	11.78±5.08 c	14.63±8.16 d	36.59±11.4 b	13.0±10.2 bc	16.89±11.74 a	4.86±1.17 a	2.22±0.81 a
	50	NM	3.64±4.59 ab	1.95±1.93 ab	8.07±10.47 a	1.91± 2.52 a	2.61±2.10 a	47.1±16.3 bc	34.33±3.26 e
		M	8.22±0.53 bc	10.11±0.3 cd	36.34±11.1 b	22.14±2.67 c	11.71±8.12 a	6.47±3.91 a	4.01±0.20 ab
	100	NM	2.35±0.41 a	0.72±0.77 a	1.48± 1.06 a	1.11±0.56 a	0.273±0.08 a	53.05±2.7 c	40.71±1.00 f
		M	6.95±7.0 abc	10.09±3.3 bc	13.87±8.62 a	6.63±3.10 a	30.03±8.78 a	20.73±9.8 bc	11.71±3.9 cd
	200	NM	5.32±0.5 abc	4.16±1.07abc	9.87±2.74 a	3.36±0.56 a	2.99±3.55 a	31.74±3.37 b	42.55±6.01 e
		M	10.16±2.0 bc	8.15±1.23 bc	31.27±5.73 b	10.76±3.03 a	5.42±2.78 a	18.39±6.43 a	16.19±1.76 d
	Two-Way ANOVA								
	All (df = 7)		2.92*	6.01***	8.65***	4.63**	23.53 ***	16.63***	48.05***
	HM (df = 3)		2.64n.s.	1.09n.s.	5.67**	5.62**	24.89***	25.39***	42.07***
	AM (df = 1)		12.3 **	33.01***	40.14***	7.11*	15.75***	23.57**	174.39***
	HM*AM (df = 3)		0.068n.s.	1.92n.s.	1.12 n.s.	2.81n.s.	24.76***	5.54**	11.92***

Means with similar letters are not significantly different according to Duncan's multiple range comparisons and bi-directional ANOVA

* Significant at *p*<0.05

** very significant at *p*<0.01

*** very high significant at *p*<0.001; AM: arbuscular mycorrhiza

The accumulation of Zn in wheat shoot was significantly (*p*<0.05) increased as the soil Zn level increased (Table 4). Mycorrhizal wheat recorded a reduced Zn level in shoot significantly (*p*<0.05). The concentration of accumulated Zn in NM shoots increased significantly at levels 0, 50 and 100 mg kg^-1^ of soil added Zn, while decreased at 200 mg kg^-1^ of Zn. In the mycorrhizal shoots, the Zn content ranges from 10.09±5.64 to 20.89±5.77% at 0 and 200 mg kg^-1^ of Zn, respectively. NM wheat treated with 200 mg kg^-1^ of Zn had a significantly higher (*p*<0.05) accumulation rate of in grains in comparison with mycorrhizal wheat. EDX spectra recorded lower accumulation of Zn in M wheat grains than that of NM (Table 4).

**Fig 3:**
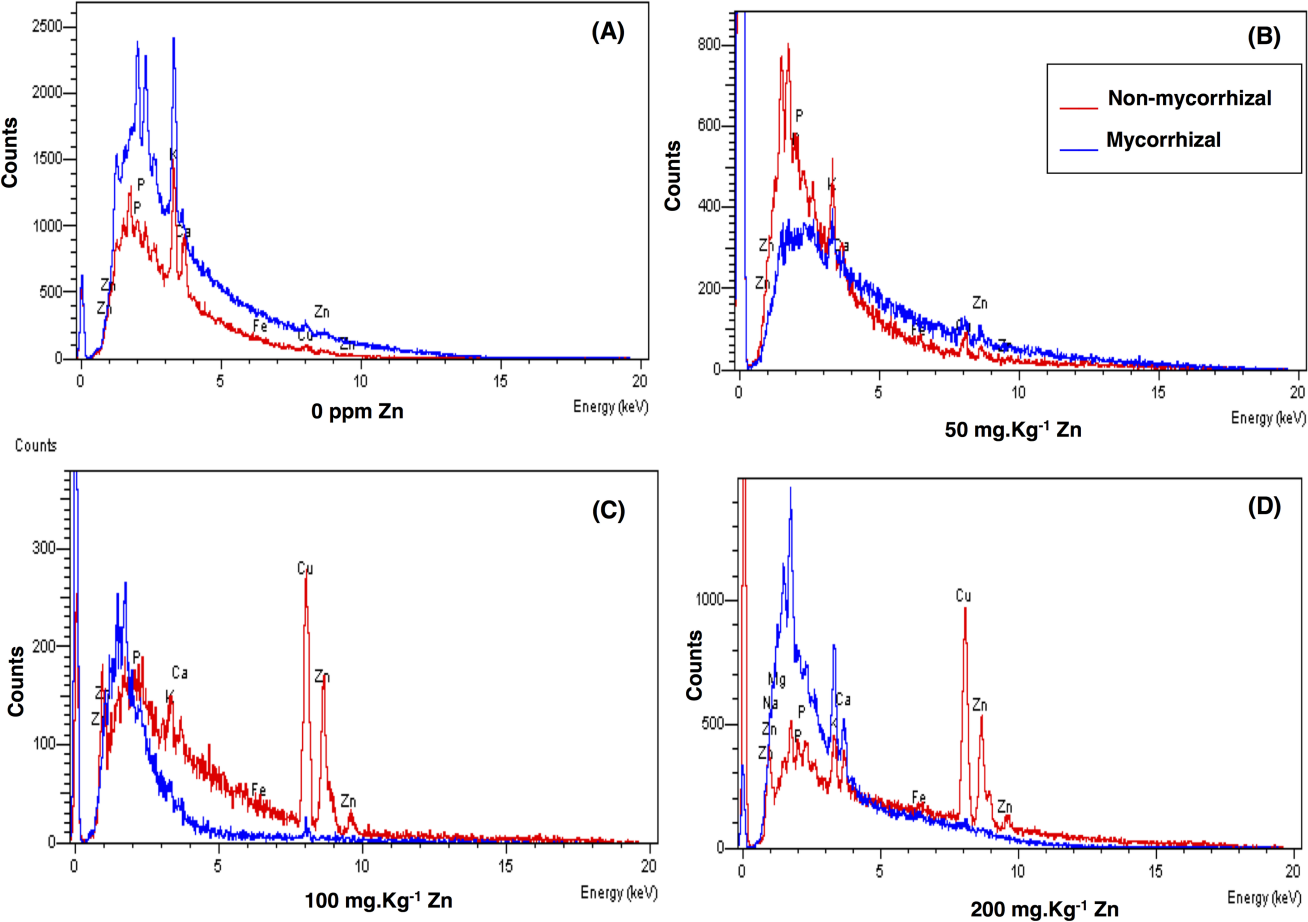
EDX spectra of wheat grains grown on soils contaminated with different levels of Zn, (A) 0 mg kg^-1^ Zn; (B) 50 mg kg^-1^ Zn; (C) 100 mg kg^-1^ Zn and (D) 200 mg kg^-1^ Zn.

The metal uptake of Zn increased in wheat (NM, M) with increasing amounts of Zn added to the soil, however, the bioaccumulation factor and phytoextraction efficiency decreased with increasing the amount of Zn. Inoculation of mycorrhizae lowered the metal uptake of Zn significantly (*p*<0.05) as compared to the NM wheat at all Zn levels (Table 5). The data plotted in Fig 3 indicated that the Zn translocation was increased at all Zn levels as compared to the control with special reference to non-mycorrhizal wheat. Both AM inoculation and Zn addition had a significant effect on metal uptake, bioaccumulation factor, and interactions between them were also significant in case of metal uptake (Table 5), however, Zn addition had a significant effect on the phytoextraction efficiency. Zn treatment of 0 and 200 mg kg^-1^ induced a significant increase in the metal uptake from 228.53±41.53 to 755.32±198.7 g pot^-1^, and from 213.83±27.24 to 666.77±67.18 g pot^-1^ in NM and M wheat, respectively. In addition, the phytoextraction decreased significantly with Zn concentration of M and NM wheat, however, in mycorrhizal wheat phytoextraction decreased markedly (Table 5).

**Table 5:** The phytoextraction efficiency (%), metal uptake (g pot^-1^), and bioaccumulation factor (BCF) of Zn of wheat subjected to zinc treatments. Data presented as the mean of three replicates followed by the standard deviation (Mean±SD).

Treatment		Zinc		
Zn (mg kg^-1^)	Inoculation.	Phytoextraction	Metal uptake	Bioaccumulation factor (BCF)
**0**	**NM**	**9.07** ± 1.64 ^b^	**228.53** ± 41.33 ^a^	**14.19** ± 1.79 ^c^
	**M**	**8.49** ± 1.08 ^b^	**213.83** ± 27.24 ^a^	**10.80** ± 1.82 ^b^
**50**	**NM**	**0.96** ± 0.27 ^a^	**745.29** ± 210.5 ^c^	**1.98** ± 0.14 ^a^
	**M**	**0.37** ± 0.09 ^a^	**288.50** ± 68.11 ^ab^	**0.44** ± 0.01 ^a^
**100**	**NM**	**0.57** ± 0.13 ^a^	**869.26** ± 205.7 ^c^	**1.34** ± 0.08 ^a^
	**M**	**0.36** ± 0.07 ^a^	**547.24** ± 103.2 ^abc^	**0.46** ± 0.08 ^a^
**200**	**NM**	**0.25** ± 0.07 ^a^	**755.32** ± 198.7 ^c^	**0.68** ± 0.04 ^a^
	**M**	**0.22** ± 0.02 ^a^	**666.77** ± 67.18 ^bc^	**0.27** ± 0.02 ^a^
**ANOVA**				
**F**_**Zn**_		209.06 **	17.225**	248.18 **
**F**_**AMF**_		1.51 NS	15.73**	17.624 **
**F**_Zn__*__AMF_		0.236 NS	3.394*	3.123 NS

Statistical analysis was performed by two-way ANOVA

NS: not significant (*p*>0.05)

*—significant (*p*<0.05)

**—highly significant (*p*<0.01) according to the MSTATC test.

The nutrient content (P, S, K, Ca, Fe, Cu, and Zn) in the roots was presented in Table 4. A slight decrease in the concentration of P, K, and S in the NM wheat treated with Zn. There were significant differences in P concentration between M and NM wheat treated with Zn in which M: NM ratio for P ranged from 1.94 to 6.14 for all cases. Inoculation of native mycorrhizae increased the concentration of P in the roots significantly of which 5.57±1.54, 8.62±1.57, 7.09±1.82 and 10.56±1.51% at all levels of Zn (0, 50, 100, and 200 mg kg^-1^) respectively. While, in the NM roots P reached a level of 2.87±1.85, 3.11±1.02, 2.22±2.61, 1.72±0.06% at the same Zn levels, respectively (Table 4). Mycorrhizal inoculated wheat showed higher Ca content that NM in roots at Zn level of 0, 50 and 100 mg kg^-1^ (Table 4). Mycorrhizal inoculation induced significant differences in Cu accumulation in roots. The Cu accumulation was decreased in M that NM roots at Zn levels of 50, 100 and 200 mg kg^-1^; respectively. The concentration of Fe in the M roots increased significantly against corresponding NM ones and recorded a level of 42.56±6.65, 54.52±3.27, 48.59±5.56, 47.91±2.74% at 0, 50, 100 and 200 mg kg^-1^ Zn, respectively.

A significant maximum level of P (16.96±2.08%) in shoots was recorded in wheat inoculated with *F*. *geosporum* at 50 mg kg^-1^ of Zn, whereas at 100 and 200 mg kg^-1^ Zn reached level of 12.41±1.96% and 12.47±4.06%, respectively. In NM wheat shoots, total P uptake decreased recording a level of 5.80±3.14, 4.21±1.69, 2.25±0.20 and 2.13±1.92% with the increasing of Zn levels from 0 to 200 mg kg^-1^, respectively. The M: NM ratio for P uptake ranged from 1.11 to 5.85 for all Zn treatments. A slight decrease in concentration of Fe and S in M wheat shoots at different Zn levels was observed (Table 4). A non-significant difference was observed in Cu concentration between NM and M wheat shoots with 0 level Zn. Cu concentration decreased significantly in M wheat shoots than NM at different Zn levels 50, 100, 200 mg kg^-1^, respectively. The concentration of K in shoots decreased significantly (*p*<0.05) in NM wheat (Table 4) reached 6.98±1.11% at high Zn level. Likewise, the Ca content of shoots of non-mycorrhizal plants reached 20.88±3.72, 2.45±0.52, 0.71±0.74 and 3.50±0.92% at 0, 50, 100 and 200 mg kg^-1^ Zn, respectively. While in M wheat shoot, it varied from 26.79±10.8 to 11.23±6.30% to 0 and 200 mg kg^-1^ Zn, respectively (Table 4).

Zinc treatments stimulated *F*. *geosporum* root colonization intensity (M%), root colonization frequencies (F%), and arbuscular development (A%) in wheat roots in different growth stages (Table 6). Microscopic examination revealed that NM roots were not colonized by AM fungi (S1 Fig). Mycorrhizal colonization in terms of F%, M%, and A% were higher at all levels Zn treatments compared to 0 Zn level in the vegetative stage after 45 days (Table 6). However, in the flowering stage (90 days), mycorrhizal colonization increased at all levels of Zn treatments compared to the vegetative stage. At 0 Zn treatment the F% was ranged from 36±2.1 to 62.3±2.0%, while A% increased non-significantly from 16.7±4.1 to 51.4±4.8% in vegetative and maturity stages, respectively (Table 6). The intensity of mycorrhizal colonization was variable and strongly influenced by Zn treatments. The results in Table 6 indicate that the increase in Zn concentrations induced a dose dependent fluctuating effect on mycorrhizal colonization.

**Table 6:** Influence of different levels of Zn on the mycorrhizal colonization of wheat. F% Frequency of mycorrhizal root segments, M% intensity of mycorrhizal colonization in the root, A% arbuscular frequency in the roots. The data represented are an average of three replicates (mean ± SD) followed by one standard deviation.

Growth stages		Treatments with Zn (mg kg^-1^)				R Coeff.	ANOVA (F-ratio)
		0	50	100	200		
**Vegetative** (45 days after germination)	**F %**	36.0±2.10^a^	48.0±5.03^b^	52.0±4.36^b^	53.0±1.11^b^	0.78**	15.6**
	**M %**	22.3±2.85^ab^	29.3±4.34^b^	19.0±3.61^a^	16.0±3.36^a^	-0.71**	10.9**
	**A %**	16.7±4.10^a^	19.0±5.02^a^	13.0±3.61^a^	8.0±5.68^a^	-0.32n.s.	0.7 n.s.
**Flowering** (70 days after germination)	**F %**	42.0±4.19^a^	62.0±4.58b	67.0±3.90^b^	59.0±10.8^b^	0.47n.s.	18.7**
	**M %**	34.0±3.01^a^	52.0±6.00^b^	58.0±7.69^b^	49.0±3.88^b^	0.47n.s.	14.8**
	**A %**	38.0±4.44^a^	36.3±2.82^a^	42.3±5.41^a^	35.6±3.60^a^	0.02n.s.	0.7 n.s.
**Maturity** (119 days after germination)	**F %**	62.3±2.00^a^	82.3±3.25^bc^	85.0±5.13^c^	75.0±6.25^b^	0.33n.s.	27.5**
	**M**	55.2±1.73^a^	58.9±3.61^a^	61.3±2.08^a^	53.4±2.06^a^	-0.05n.s.	0.8n.s.
	**A %**	51.4±4.84^a^	53.8±6.25^a^	59.4±3.61^a^	59.3±9.50^a^	0.49n.s.	1.3n.s.

Means in the same row with similar letters are not significantly different according to the Duncan and ANOVA multiple range comparisons. Spearman rank correlation was performed against different treatment concentrations representing the Spearman correlation coefficient and the significance of two tails.

* Significant at *p*<0.05

** significant at *p*<0.01

n.s. non-significant at *p*>0.05.

**Fig 4:**
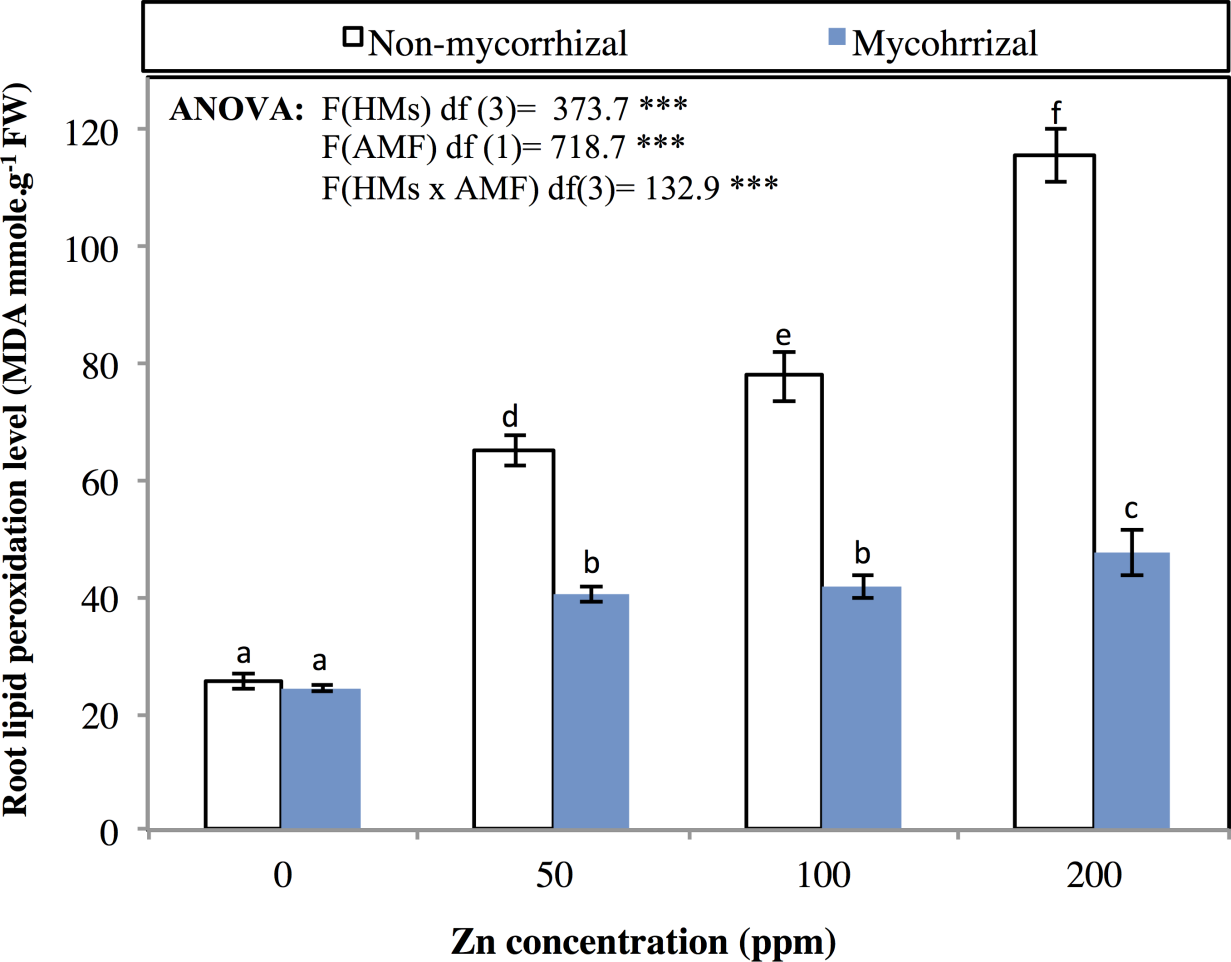
Influence of mycorrhizal colonization on lipid peroxidation as MDA (mmole g^-1^ FW). **The data represented are an average of five replicates; the error bars represent a standard error for the means.** ANOVA was performed to assess differences between treatment groups, followed by Duncan's multiple-rank comparisons. Media with similar letters are not significantly different according to Duncan's multiple range comparisons.

Lipid peroxidation was estimated in terms of Malondialdehyde (MDA) and its content in NM and M wheat increased significantly (p<0.05) with the increasing zinc level of 0 to 50, 100, 200 mg kg^-1^ (Fig 4). The level of lipid peroxidation ranged from 25.38±1.34 to 115.52±4.6 in non-mycorrhizal wheat; in mycorrhizal plants ranged from 24.23±0.43 to 47.35±3.78 (Fig 4). Mycorrhizal inoculation with *F*. *geosporum* significantly (p<0.05) decreased cellular lipid peroxidation in wheat in terms of MDA revealed by Duncan’s multiple range comparisons.

## Discussion

Our data demonstrated that arbuscular mycorrhizae were successfully colonized and the frequency of root colonization was increased with zinc treatments. The photosynthetic pigment fractions were significantly affected by Zn treatments and mycorrhizal inoculation, where in all treatments, the content of the photosynthetic pigment fractions decreased as the Zn concentration increased in the soil. The dry weight of mycorrhizal wheat increases at all levels of Zn compared to the 0 mg Kg^-1^ Zn level. Inoculation with *F*. *geosporum* improved the growth and yield of wheat under higher levels of Zn and can be applied to improve soils contaminated with zinc. The decrease in chlorophyll content, as an index to assess photosynthesis, in wheat plants as a result of Zn deficiency has also been reported by several authors and in other plant species [72]. The increase in photosynthetic pigments as a result of mycorrhizal colonization was also supported by Sánchez-Blanco et al. [73] and Wu and Xia [74]. The present results indicate that the application of AM helps the plants to counter photodamage and the photoinhibition of pigments under stress conditions. Increased carotenoid content, as a protective scenario, can directly deactivate singlet oxygen (^1^O_2_) to protect the photosynthetic apparatus against photoinhibition damage and can also extinguish excited ^3^Chl*, i.e. triple chlorophyll status [75,76]. The higher content of chlorophyll in mycorrhizal (M) plants than non-mycorrhizal (NM) plants has sometimes been related to a higher rate of photosynthesis or due to the increase of N and Mg contents (main components of chlorophyll molecules) in the mycorrhizal plants [77].

The concentrations of minerals in shoots, roots, and grains were influenced to a great extent by the treatment with zinc and inoculation with the native mycorrhizal fungus *F*. *geosporum*. Treatment with Zn in the soil increased the concentrations of Zn and Cu in the root; the tissues of the shoots and the grains of all the plants studied, while the treatment with zinc decreased the other minerals (P, S, K, Ca and Fe) in the tissues of shoots, roots and grains. Zn and P are observed to interact and may interfere with the availability and utilization of others [78]. The high Zn uptake efficiency is inversely proportional to the P uptake in the root system and may imply a high rate of Zn transport from the roots to the shoot through the xylem, which may hinder the translocation of P from the roots to shoots [78, 79]. Our conclusion is in agreement with other studies carried out by Samreen et al. [78] and Zhu et al. [79]. The application of zinc may have opposite influence on iron content (Fe) and absorption in plants. The competitive interaction with zinc can cause the decrease of Fe and other mineral ions absorption in the root system. Similar findings for the competitive interaction between Zn and other minerals were reported by Loneragan and Webb [80], Rajaie et al. [81] and Samreen et al. [78]. Zn strongly affects the metabolic function of iron in plants; excess of one can depress the absorption of others.

Shoot of mycorrhizal wheat had higher concentrations of P, K, Ca, S and Fe than non-mycorrhizal (NM) plants; however, Cu and Zn were present in low concentrations in root and shoot systems of mycorrhizal wheat. These findings are also supported by several reports in which mycorrhizal inoculation can increase the plant growth under different Zn levels by increasing nutrient uptake; e.g. P [82–84]; K [85, 86]. However, Cu and Zn were lower in roots, shoots and grains of mycorrhizal plants than non-mycorrhizal [87, 88]. While, in other studies, Zn were found to increase and accumulate in the roots of AM inoculated plants [89, 90] and copper [83]. Reports on the accumulation of metals at high levels indicate that there are differences in metal accumulation and inter-plant translocation depending on the mycorrhizal fungal species, root system density, host-plant, soil characteristics, metals and metals availability [91, 92].

Arbuscular mycorrhizal inoculation is recognized to alleviate the abiotic stress of heavy metals in plants [61, 93, 94]. Navarro-Ródenas et al. [95] noted that inoculation of *Citrus* root stocks with AM and irrigated with saline water, showed a significant increase in the growth of inoculated plants than NM *Citrus* irrigated with non-saline water. The mechanism by which the inoculation of arbuscular mycorrhizae increases the tolerance of the plants to diverse abiotic tensions, salinity, drought and heavy metal stress is mostly nutritional mechanism [93, 95–97] demonstrated that both P fertilization and AM inoculation of plants significantly improved plant growth in soils contaminated with HMs. Aforementioned authors concluded that AM fungi increase plant tolerance to heavy metal stresses mainly through phosphorus nutrition. Non-nutritional mechanisms by which AM fungi increase plant tolerance to abiotic stress e.g. drought implies: changes in hormone levels, improved soil water status, delayed soil drying, improvement of hyphal soil, increased photosynthetic rate and accumulation of various compatible osmolytes [96–98]. Immobilization of heavy metals in mycorrhizal structures and biomass (e.g. in the vesicle, cell wall and glycoprotein (glomalin)) can be considered as a non-nutritional mechanism by which mycorrhizal inoculation improve the plant tolerance to abiotic stress of heavy metals [61].

## Conclusions

The present study focused on the bioremediation of soils contaminated with zinc by using a strategy that included the selection of a native mycorrhizal taxon that was successfully colonized with wheat roots. Mycorrhizal inoculation enhanced fresh weight and dry shoots of mycorrhizal plants under different Zn concentrations. The mycorrhizal inoculation of *T*. *aestivum* improved the photosynthetic pigment affected by Zn treatments and mycorrhizal colonization decreased as soil Zn increased. On the other hand, the level of minerals of shoots, roots, and grains was greatly influenced by Zn-treatment and by inoculation with *F*. *geosporum*. Treatment with Zn in the soil increased Cu and Zn concentrations in the root, shoot and grains, however, other minerals (P, S, K, Ca and Fe) decreased. Inoculation of wheat with AM fungi significantly reduced the accumulation of Zn in shoots and grains that diminished the Zn translocation in shoots and grains of wheat. In conclusion, inoculation with a native mycorrhizal taxon (*F*. *geosporum)* improves yields of wheat under higher levels of Zn and is possible to be applied for the improvement of zinc contaminated.

### Ethics statement

No specific permits were required for the described field studies, as the location where samples were collected is not privately owned or protected. We confirm that the field studies did not involve endangered or protected species. All relevant data are within the paper and its Supporting Information files.

## Supporting information

S1 Fig (A-F) Mycorrhizal colonization of wheat under high levels of zinc, (A-B) Vesicles; (C-D) Arbuscules; (E) Hyphal coils; (F) Extraradical hyphae.
(TIF)

## Abbreviations

HMs: heavy metals
MDA: Malondialdehyde
AMF: arbuscular mycorrhizal fungi
NM: non-mycorrhizal
M: mycorrhizal
NS: none-significant
SEM: Scanning electron microscope
EDX: energy dispersive X-ray
BCF: bioaccumulation factor
