# Supplementary figures and images for: Arbuscular mycorrhizal strategy for zinc mycoremediation and diminished translocation to shoots and grains in wheat
Source: PLoS One. 2017 Nov 16;12(11):e0188220. doi: 10.1371/journal.pone.0188220 (PMC5690681; doi:10.1371/journal.pone.0188220)

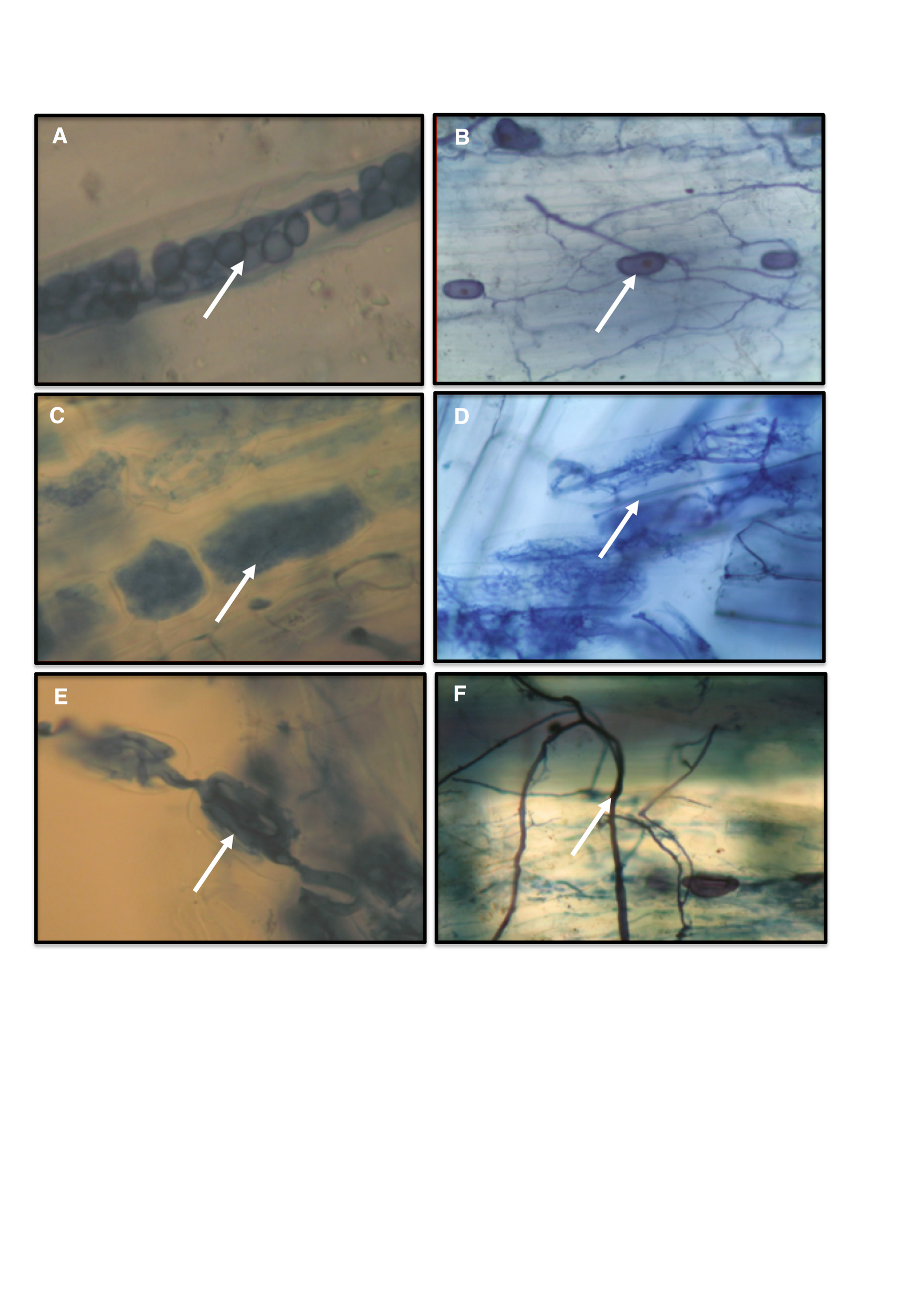

Supplement: S1 Fig — (A-F) Mycorrhizal colonization of wheat under high levels of zinc, (A-B) Vesicles; (C-D) Arbuscules; (E) Hyphal coils; (F) Extraradical hyphae. (TIF) [file pone.0188220.s001.tif]
